# Impact of Sarcopenia on Survival and Clinical Outcomes in Patients With Liver Cirrhosis

**DOI:** 10.3389/fnut.2021.766451

**Published:** 2021-10-21

**Authors:** Mirabela-Madalina Topan, Ioan Sporea, Mirela Dănilă, Alina Popescu, Ana-Maria Ghiuchici, Raluca Lupuşoru, Roxana Şirli

**Affiliations:** ^1^Department of Gastroenterology and Hepatology, “Victor Babeş” University of Medicine and Pharmacy, Timişoara, Romania; ^2^Advanced Regional Research Center in Gastroenterology and Hepatology of the “Victor Babeş” University of Medicine and Pharmacy, Timişoara, Romania; ^3^Department of Functional Science, Center for Modeling Biological Systems and Data Analysis, University of Medicine and Pharmacy Victor Babeş, Timişoara, Romania

**Keywords:** sarcopenia, liver cirrhosis, clinical outcomes, survival, handgrip strength, skeletal muscle index

## Abstract

**Background:** Sarcopenia is now recognized more and more as a biomarker with poor outcomes in cirrhotic patients.

**Aims:** The purpose of this study was to investigate the prevalence of sarcopenia in patients with liver cirrhosis and prospectively investigate the association between sarcopenia and different complications and its impact on survival.

**Material and Methods:** This prospective study included patients with liver cirrhosis admitted to our department from 2018 to 2020. Sarcopenia was assessed according to EWGSOP2 criteria, incorporating low Handgrip strength (<27 kg for men and <16 kg for women) with low skeletal muscle index evaluated by CT (<50 for men and <39 for women). Associations between sarcopenia and portal hypertension-related complications, infectious complications, and risk of hepatocellular carcinoma, the number of in-hospital days, 30-day readmission, and survival over the next 6 and 12 months were analyzed.

**Results:** A total of 201 patients were enrolled in the study, 63.2% male, mean age 61.65 ± 9.49 years, 79.6% Child-Pugh class B and C. The primary etiology of liver cirrhosis was alcohol consumption (55.2%). The prevalence of sarcopenia was 57.2 %, with no significant differences between the male and female groups. Significant associations were found between sarcopenia and portal hypertension-related complications, infectious complications, and risk of hepatocellular carcinoma. In multivariate analysis, sarcopenia was assessed as a risk factor alone, increasing the risk for ascites 3.78 times, hepatocellular carcinoma by 9.23 times, urinary tract infection by 4.83 times, and spontaneous peritonitis 2.49 times. Sarcopenia was associated with more extended hospital stay and higher 30 days readmission. Six months and 1-year survival were reduced in the sarcopenia group than in the non-sarcopenia group (*p* < 0.0001).

**Conclusion:** Sarcopenia is a common complication of liver cirrhosis and associates with adverse health-related outcomes and poor survival rates.

## Introduction

During the last years, various scientific groups attempted to develop different definitions for sarcopenia. The European Working Group on Sarcopenia in Older People (EWGSOP2) (1) updated in 2019 their previous definition of sarcopenia, which is now defined as a muscle disease (low muscle quantity and quality) associated with low muscle strength. Liver cirrhosis is one of the most representative chronic diseases, which can be complicated by sarcopenia. Clinical practice guidelines of the European Association for the study of the Liver (EASL) (2) and the European Society for Clinical Nutrition and Metabolism (ESPEN) (3) recommend screening for sarcopenia as its early recognition is a critical aspect of the care of these patients.

The prevalence of sarcopenia in Liver Cirrhosis is around 23–60% (4), but this percentage depends on the severity of the underlying liver disease and the diagnostic tools and criteria utilized.

Previous studies have evaluated the association between sarcopenia and higher rates of other cirrhosis complications, infections, hospital admissions, and reduced survival (5–8), but few of those studies applied the new EWGSOP2 criteria to define sarcopenia. Data are lacking, whether the 2010 or 2019 diagnosis criteria better predict complications and poor prognosis.

Therefore, the present study aimed to evaluate the prevalence of sarcopenia in patients with liver cirrhosis using the 2019 sarcopenia consensus definition of EWGSOP2 and prospectively investigate the association between sarcopenia and a higher rate of complication and poor survival.

## Materials and Methods

### Study Design and Population Selection

This is a prospective, observational study, carried out in a tertiary Department of Gastroenterology and Hepatology, from January 2018 to December 2020 on 201 patients with liver cirrhosis.

Liver cirrhosis diagnosis was based on physical examination, abdominal ultrasound, laboratory tests, ultrasound-based elastography, upper endoscopy, and radiological evidence. Child Pugh's score and the Model for End-Stage Liver Disease (MELD) score were used for liver function assessment.

The present study includes 201 patients. Based on the following inclusion criteria: patients with liver cirrhosis older than 18 years and availability of a diagnostic reference standard method (Contrast-enhanced Computer Tomograph). The exclusion criteria were: patients with any factors that could independently influence sarcopenia such as Human Immunodeficiency Virus, tuberculosis, obstructive pulmonary disease, chronic renal failure, congestive heart failure, neuromuscular disorders, inflammatory bowel disease, other malignancies than hepatocellular carcinoma.

Data collected from medical charts included: age, gender, etiology, albumin, INR, Sodium, Thrombocytes, Child-Pugh score, MELD score, presence of ascites, presence of esophageal varices, upper gastrointestinal bleeding (upper GI bleed), urinary tract infection (UTI), Pneumonia, spontaneous bacterial peritonitis (PBS), hepatic encephalopathy (HE), hepatorenal syndrome (SHR), Hepatocellular carcinoma (HCC), 30-day readmission, length of hospitalization, 6 months and 1-year mortality.

The study protocol was approved by the local Ethical Committee and was performed in accordance with the Helsinki Declaration of 1975, after informed consent to participate in the study was obtained from every patient.

### Anthropometric Measurements

#### Handgrip Strength (HGS)

Dominant handgrip strength was measured using a Jamar dynamometer. The patient was examined while sitting down with the elbow flexed at 90° and the arm along the body or in dorsal position with the elbow supported and the head at 30°. Each patient used the dominant hand and performed the test three times with a pause of 10–30 s between the tests. The highest record value was used. All values were recorded in kilograms.

#### Skeletal Muscle Index (SMI)

Computer Tomography (CT) images for cross-sectional skeletal muscle mass assessment were analyzed at the level of lumbar 3 by a single observer, using National Institutes of Health ImageJ software. For muscle tissue, standard attenuation values ranged from 29 to 150 Hounsfield units. The cross-sectional areas achieved were normalized for patient height, obtaining the skeletal muscle index, which is expressed as *a cross-sectional muscle area/height*^2^. The measurements were done by an experienced radiologist.

### Diagnosis of Sarcopenia

Sarcopenia was defined based on the EWGSOP2 criteria using the combination of low SMI and low HGS with stratification of gender and age-specific cut-off values. Table 1 outlines the cut-offs used for SMI and HGS (1).

**Table 1:** Cut-offs values used to define sarcopenia.

	**HGS**	**SMI**
Male	<27 kg	<50 *cm*^2^/*m*^2^
Female	<16 kg	<39 *cm*^2^/*m*^2^

### Statistical Analysis

The statistical analysis was performed using MedCald software for windows (MedCalc Software, version 19.3.1, Ostend, Belgium). Categorical data were described as number and percentage, and continuous data were described as mean and standard deviation. Skewed data were described as median and interquartile line. For correlation analysis of categorical data Spearman's rho and Kendall's tau-b were used. A 5% significance level was considered. Predictors for sarcopenia were assessed using regression analysis. A risk analysis was made.

## Results

### Patients Characteristics

Two hundred and one patients fulfilled the inclusion criteria and were included in the analysis, mean age 61.65 ± 9.49 years. The male gender was predominant 63.2%. Regarding etiology, more than half (55.2%) had alcoholic cirrhosis, 24.8% hepatitis C virus (HCV) cirrhosis, 8.9% hepatitis B virus (HBV) cirrhosis, 10.9% other etiologies. According to the Child-Pugh Classification: 20.4% were A class, 40.8% were B, and 38.81% were C. Table 2 shows the baseline characteristics of the study population.

**Table 2:** Baseline characteristics of the study population.

**Parameter**	**Values**
Age [years] (mean ± SD)	61.65 ± 9.49
• <40 years • 40–60 years •>60 years	• 1 (0.5%) • 114 (56.7%) • 86 (42.7%)
Gender–Males *n* (%)	96 (61.5%)
Child-Pugh classification	
• A • B • C	• 41 (20.4%) • 82 (40.8%) • 78 (38.8%)
Mean Child Pugh score (points)	8.79 ± 2.24
Mean MELD score (points)	16.56 ± 7.59
Ascites *n* (%)	
• Absent • Present	• 44 (21.8%) • 157 (78.8%)
Etiology of cirrhosis *n* (%)	
• Hepatitis B • Hepatitis C • Alcohol abuse • Other	• 18 (9%) • 50 (24.9%) • 111 (55.2%) • 22 (10.9%)

### Prevalence of Sarcopenia

According to the EWGSOP2 criteria, the prevalence of sarcopenia in our overall cohort was 57.2% (*p* < 0.0001). 108/160 patients (67.5%) in the decompensated group had sarcopenia, while only 7/41 patients (17.07%) were sarcopenic in the compensated group.

There were no differences between gender concerning the prevalence of sarcopenia, 76 male patients with sarcopenia vs. 39 female patients with sarcopenia, *p* = 0.37.

### Sarcopenia and Clinical Outcomes and Survival Rates

When comparing the two study groups, we found significant differences between the sarcopenic group vs. the non-sarcopenic group regarding albumin level, MELD score, Child-Pugh score, sodium level, INR level, and hospitalization days (*p* < 0.05). We also found differences in proportions between the two groups regarding hepatic encephalopathy rate, ascites rate, HCC rate, urinary tract infection rate, hepato-renal syndrome rate, esophageal varices, and 6 months and 1-year mortality (*p* < 0.05) (Table 3).

**Table 3:** Comparation between sarcopenic and non-sarcopenic patient's characteristics.

**Parameter**	**Overall**	**Sarcopenia**	**Normal weight**	***p*-value**
Age	61.65 ± 9.49	61.33 ± 9.34	62.09 ± 9.78	0.57
Gender (male)	127 (63.2%)	76 (59.8%)	51 (40.2%)	0.37
MELD score	16.56 ± 7.59	18.69 ± 7.74	13.72 ± 6.39	<0.0001
Child Pugh score	8.79 ± 2.24	9.71 ± 1.90	4.56 ± 2.07	<0.0001
SMI	44.44 ± 6.76	42.38 ± 6.21	47.33 ± 6.53	<0.0001
HGS	22.10 ± 8.55	17.84 ± 5.48	28.04 ± 8.47	<0.0001
Albumin	2.53 ± 0.72	2.27 ± 0.61	2.89 ± 0.71	<0.0001
Sodium	136.3 ± 5.38	134.95 ± 6.00	138.17 ± 3.74	<0.0001
INR	1.47 ± 0.38	1.54 ± 0.41	1.38 ± 0.31	0.002
Thrombocytes	124 (22-552)	129.6 (22-156)	148 (47-552)	0.35
Hepatic Encephalopathy	61 (30.3%)	50 (81.9%)	11 (18.9%)	<0.001
Ascites	157 (78.1%)	109 (69.4%)	48 (30.6%)	<0.0001
Esophageal varices	157 (78.1%)	97 (61.7%)	60 (38.3%)	0.01
Hepato-renal syndrome	7 (3.4%)	7 (100%)	0	0.04
Upper GI bleeding	76 (37.8%)	49 (64.4%)	27 (35.6%)	0.10
Hepatocellular carcinoma	65 (32.3%)	54 (83.0%)	11 (17.0%)	<0.0001
Urinary tract infection	61 (30.1%)	46 (75.4%)	15 (24.6%)	0.0005
Spontaneous peritonitis	40 (19.9%)	35 (87.5%)	5 (12.5%)	<0.0001
Pulmonary infections	34 (16.9%)	24 (70.5%)	10 (29.5%)	0.07
Hospitalization days	10.19 ± 6.03	13.36 ± 5.39	5.98 ± 3.90	<0.0001
30 days readmission	75 (37.5%)	64 (85.3%)	11 (14.7%)	<0.0001
6 months mortality	62 (30.8%)	56 (90.3%)	6 (9.7%)	<0.0001
1 year mortality	122 (60.6%)	96 (78.6%)	26 (21.4%)	<0.0001

While not statistically significant, a larger percentage of sarcopenic patients presented upper gastrointestinal bleeding and pulmonary infections vs. non-sarcopenic patients (64.4 vs. 35.6%, 70.5 vs. 29.5%, respectively).

As shown in Table 4, a correlation analysis of different factors was made, and various associations with sarcopenia were found. For example, regarding albumin and sodium levels, if albumin or sodium level decreases, sarcopenia chances increase, *p* < 0.0001. For MELD score, Child-Pugh Score, INR level, and length of hospitalization days, if the values are increasing, the chance of patients being sarcopenic is increasing as well.

**Table 4:** Regression and correlation analysis of factors involved in sarcopenia.

**Parameter**	**Correlation coefficient**	***p*-value**
Albumin	−0.42	<0.0001
Encephalopathy	0.33	<0.0001
Hospitalization days	0.59	<0.0001
Ascites	0.46	<0.0001
30 days readmission	0.44	<0.0001
MELD score	0.32	<0.0001
Hepatocellular carcinoma	0.36	<0.0001
Upper gastrointestinal bleeding	0.11	0.10
INR	0.21	<0.0001
Urinary tract infection	0.24	0.0004
Sodium	−0.29	<0.0001
Spontaneous peritonitis	0.30	<0.0001
Hepato-renal syndrome	0.16	0.01
Age	−0.03	0.57
Pulmonary infections	0.12	0.07
Esophageal varices	0.17	<0.01
Child Pugh Score	0.47	<0.0001
6 months mortality	0.45	<0.0001
1 year mortality	0.53	<0.0001

Other factors associated with sarcopenia were hepatic encephalopathy, ascites, 30 days readmission rate, hepatocellular carcinoma, spontaneous peritonitis, urinary tract infection, hepato-renal syndrome, presence of esophageal varices, and 6 months and 1 year mortality (*p* < 0.05).

In multivariate analysis, sarcopenia was assessed as a risk factor alone, increasing the risk for ascites 3.78 times, hepatocellular carcinoma by 9.23 times, urinary tract infection by 4.83 times, and spontaneous peritonitis 2.49 times, as shown in Table 5.

**Table 5:** Multivariate logistic regression analysis of factors associated with sarcopenia.

**Parameter**	**Odds ratio**	**95% CI**	***p*-value**
1 year mortality	0.73	0.21 - 2.47	0.60
6 months mortality	1.37	0.40–4.73	0.60
30 days readmission	1.61	0.42–6.05	0.47
Ascites	3.78	0.85–16.86	0.04
Encephalopathy	0.68	0.20–2.33	0.05
Hepatocarcinoma	9.23	2.42–35.16	0.0001
Hospitalization days	1.35	1.15–1.58	0.60
INR	0.70	0.18–2.66	0.31
Urinary tract infections	4.83	1.77–13.22	0.002
MELD score	0.59	0.88–1.03	0.15
Sodium	0.92	0.83–1.03	0.18
Spontaneous peritonitis	2.49	0.63–9.77	0.03
Child Pugh score	1.01	0.69–1.76	0.93
Hepatorenal syndrome	5.30	0.75–6.80	0.42
Esophageal varices	2.30	0.80–6.57	0.11

## Discussion

### Sarcopenia Assessment

Sarcopenia is a frequent complication of cirrhosis. There is a lack of consensus concerning which criteria to use to define sarcopenia in patients with liver cirrhosis. In 2019, EWGSOP2 (1) updated the definition of sarcopenia and provided consensus criteria in which low muscle mass and low muscle strength were required for the diagnosis. In an article published in 2020 by Traub et al. (9), a comparison between the 2010 and 2019 EWGSOP criteria was made, and the results showed that sarcopenia is less often diagnosed when using the 2019 criteria, but according to a study conducted by Anand et al. (10), the new definition of sarcopenia best predicts mortality and clinical outcomes. Son et al. concluded in a recent review (11) that further studies are required to determine which definition of sarcopenia is the most useful for predicting poor outcomes among patients with cirrhosis.

As only a few studies have used the combination of muscle mass and function to assess sarcopenia in patients with cirrhosis, we decided to apply this definition in our cohort of patients.

There are different diagnostic tools and tests available for the assessment of sarcopenia. In our study, to determine muscle mass, we used skeletal muscle index evaluated by Contrast-Enhanced CT, which is considered a gold standard for evaluating sarcopenia (2, 12). CT is frequently used in daily practice as a screening method for HCC, so it can also be used to assess sarcopenia. Although there are some limitations of this method regarding radiation exposure, costs, and the complexity of the measurement technique of SMI that requires radiological expertise and time, as well as a specialized software.

To assess muscle strength, we used HGS, a simple and inexpensive valuable tool in daily practice that can predict poor patients' outcomes and mortality (13).

### Prevalence of Sarcopenia

In our cohort, by applying the EWGSOP2 (1) criteria and cut-offs, sarcopenia was diagnosed in 57.2% of the patients, like the results found in similar articles and reviews from the literature (4, 11, 14). Given that the majority of our patients were Child-Pugh B and C, and the most common etiology was alcohol abuse (55.2%), the high prevalence of malnutrition in our cohort can be explained.

Although studies from the literature (14, 15) say that sarcopenia is more prevalent in male patients with cirrhosis, in our study, there is no statistical difference regarding the prevalence of sarcopenia between males and females.

### Sarcopenia and Survival

The prognosis of sarcopenic cirrhotic patients is significantly worse than that of non-sarcopenic patients, with a higher mortality rate (7, 15). In our study, there was a statistically significant difference between the sarcopenic and non-sarcopenic cirrhotic patients in terms of 6 months and 1-year mortality (*p* < 0.0001). The mortality rates at 6 months and 1 year of follow-up were significantly higher in sarcopenic cirrhotic patients (90.3 and 78.6%) than among non-sarcopenic cirrhotic patients (9.7 and 21.4%).

According to our analysis, sarcopenia is associated with more extended hospital stay and higher 30 days readmission, which is similar to the results found by Montano-Loza et al. (16). In the sarcopenic group, the average length of stay in the hospital was 13.36 ± 5.39 days, while the non-sarcopenia group had an average length of 5.98 ± 3.90 days, *p* < 0.0001.

### Impact of Sarcopenia on Cirrhosis Complications

Complications regarding portal hypertension such as ascites, presence of esophageal varices, hepatic encephalopathy, hepatorenal syndrome are reported to have a strong correlation with the presence of sarcopenia (17, 18). Our results also showed similar findings, as a strong association between all the above complications and sarcopenia was found (*p* < 0.0001). According to our study, sarcopenia increases the risk for ascites by 3.78 times.

There were no statistical differences between the two groups concerning the risk of variceal bleeding.

In our study, we found a significant correlation between sarcopenia and HCC. Using multivariate logistic regression analysis, we found that sarcopenic patients have a 9.23 higher risk of developing hepatocellular carcinoma than non-sarcopenic patients. In a recently published article in Clinical Nutrition, Feng et al. (19) also found out that cirrhotic patients recorded to have sarcopenia at baseline assessment had a significantly increased risk of developing HCC during a median follow-up of 3.6 years. However, this finding was limited to male patients.

Published data showed that sarcopenia had been associated with an increased risk of infections (15, 20). A statistically significant association between sarcopenia and urinary tract infection and spontaneous bacterial peritonitis (*p* < 0.0001) was found in our research, as sarcopenia increases the risk for urinary tract infection by 4.83 times and spontaneous peritonitis by 2.49 times.

### Sarcopenia and Other Implications

In the current study, a statistically significant difference was found between cirrhotic patients with and without sarcopenia in terms of MELD scoring; a higher MELD score was found in the sarcopenic group (mean MELD 18.69 ± 7.74 and 13.72 ± 6.39, respectively, *p* < 0.0001). We also found an association between sarcopenia and hypoalbuminemia, hyponatremia, thrombocytopenia, and higher INR levels. Similar differences were found in the study of Montano-Loza et al. (18).

### Study Limitations

The limitations of the present study were: firstly, the single-center study design, so a more extensive multicenter study will be needed to confirm our findings. Secondly, the cut-off values used for SMI and HGS to define sarcopenia are from a different population sample because predefined values for sarcopenia in patients with cirrhosis are lacking. Thirdly, the lack of cohort homogeneity, as most of the patients were Child-Pugh B and C.

Despite the limitations, our study adds a notable contribution to the epidemiology of sarcopenia in cirrhotic patients and provides useful information regarding the prognostic value of sarcopenia in patients with liver cirrhosis using the EWGSOP2 criteria.

## Conclusion

Sarcopenia is a highly prevalent complication of liver cirrhosis, and it is associated with a worsened clinical outcome, including increased hospitalization rates and reduced survival. A systematic evaluation of this common complication should be prioritized to increase the survival rate in these patients and decrease the hospitalization burden.

## Data Availability Statement

The raw data supporting the conclusions of this article will be made available by the authors, without undue reservation.

## Ethics Statement

The studies involving human participants were reviewed and approved by University of Medicine and Pharmacy Victor Babes Ethical Committee. The patients/participants provided their written informed consent to participate in this study.

## Author Contributions

RŞ, MD, and M-MT did the concept and design of the study and revised and completed the manuscript. M-MT performed the measurements, analyzed the data, drafted the manuscript, and revised and completed the manuscript. IS and AP revised and completed the manuscript. A-MG collected patient data and nutrition studies and revised and completed the manuscript. RL made statistical analyzed and revised and completed the manuscript. All authors critically revised the manuscript, approved the final version to be published, and agreed to be accountable for all aspects of the work.

## Conflict of Interest

The authors declare that the research was conducted in the absence of any commercial or financial relationships that could be construed as a potential conflict of interest.

## Publisher's Note

All claims expressed in this article are solely those of the authors and do not necessarily represent those of their affiliated organizations, or those of the publisher, the editors and the reviewers. Any product that may be evaluated in this article, or claim that may be made by its manufacturer, is not guaranteed or endorsed by the publisher.
